# Communication-efficient decentralized clustering for dynamical multi-agent systems

**DOI:** 10.1371/journal.pone.0327396

**Published:** 2025-07-29

**Authors:** Victoria Erofeeva, Oleg Granichin, Vikentii Pankov, Zeev Volkovich

**Affiliations:** 1 St. Petersburg State University, St. Petersburg, Russia; 2 Software Engineering Department, Braude College of Engineering, Karmiel, Israel; National University of Sciences and Technology NUST, PAKISTAN

## Abstract

The paper presents a decentralized, real-time clustering method designed for large-scale, distributed environments such as the Internet of Things (IoT). The approach combines compressed sensing for dimensionality reduction with a consensus protocol for distributed aggregation, enabling each node to generate compact, consistent summaries of the system’s clustering structure with minimal communication overhead. These representations are processed by a pre-trained neural network to reconstruct the global clustering state entirely without centralized coordination. Unlike traditional methods that depend on static topologies and centralized computation, this system adapts to dynamic network changes and supports on-the-fly processing. The system suits IoT applications where data must be processed locally, and immediate results are essential. Experiments on both synthetic and real-world datasets show that the method significantly outperforms baseline approaches in clustering accuracy, making it highly suitable for resource-limited, decentralized IoT scenarios.

## Introduction

While traditional machine learning excels in areas like facial recognition and biological network analysis using supercomputers, the growth of the Internet of Things (IoT) necessitates a shift. IoT devices demand real-time, local data processing and decision-making, moving away from centralized models. Identifying community structures within complex networks, particularly biological ones, remains crucial for understanding their behavior. This neediness persists and intensifies with IoT and edge computing. Consequently, there is an urgent demand for adaptable algorithms that can efficiently detect community structures in dynamic, distributed networks to optimize system performance.

These tools must be scalable and versatile, underscoring their significance in modern machine learning. The crucial question arises, should data from millions of distributed devices be funneled into a central system for analysis? While this traditional approach has been widely used, its limitations are becoming increasingly evident. Centralized processing leads to latency issues, network congestion, and significant privacy risks. In real-time applications such as autonomous vehicles that require instant decision-making for safety or industrial automation where delays can result in costly downtime relying on a distant central server is impractical. Additionally, the massive influx of data from globally dispersed IoT devices can overwhelm bandwidth, creating inefficiencies and bottlenecks.

Beyond performance concerns, centralizing sensitive data from wearables, smart home devices, and industrial sensors heightens privacy and security risks, making them attractive targets for cyber threats. Moreover, central processing models hinder real-time, localized decision-making. A decentralized approach enables local appliances to react promptly to real-time sensor data, optimizing flow and safety without relying on a central hub. The scalability challenge further underscores the need for a shift toward decentralized processing; with the rapid proliferation of IoT devices, traditional architecture struggles to keep pace. ‘’Edge computing‘’, an integral part of decentralized strategies, enables computation to occur closer to the data source, significantly reducing latency and bandwidth consumption while improving responsiveness. This shift improves performance and data privacy by reducing the need to transmit data extensively.

Ultimately, embracing distributed data processing is not just an enhancement; it is an essential transformation for the future of IoT. This paradigm shift is critical to building resilient, efficient, and secure IoT ecosystems by enabling intelligent, localized decision-making, reducing security risks, and improving scalability. Decentralized clustering offers a scalable and resilient approach to managing vast datasets in distributed systems.

Generally, a fundamental aspect of complex network analysis is community detection, which involves grouping nodes into clusters based on their connection strength, exploiting various pattern recognition techniques. Such a practice serves as a powerful tool for uncovering the organizational prototypes of complex networks and has been extensively applied across diverse scientific domains, such as online communities’ detection (see, e.g., [1,2]), the analysis of biological networks (see, e.g., [3]) and so on.

The decentralized clustering methodology arising within this general outlook is a pivotal direction in machine learning, especially when dealing with large-scale datasets that exceed a single machine’s computational and memory capacity. In distributed setups, data points are divided across multiple nodes (e.g., servers or devices), where local computations are performed. These local results are consequently combined with the construction of a global partition model. One of the primary challenges in this process is ensuring communication efficiency, namely, minimizing the data exchanged between nodes, while preserving the accuracy and quality of the final clustering outcome.

Implementing this methodology in a decentralized multi-agent system, where the network operates without a central server, appears highly effective. For example, a sparsely connected robotic network, with nodes maintaining only limited direct peer connections, optimizes for minimal communication links. This conserves resources, minimizes bandwidth consumption, and improves scalability compared to densely interconnected structures.

This paper imparts a novel approach to distributed clustering built around the ‘’cluster synchronization‘’ concept. Contrasting conventional methods that struggle to adapt to the fluid nature of real-world networks, our strategy is designed to adjust to the evolving system states dynamically. The method’s core strength is its continuous tracking and response to evolving cluster structures, which ensures relevance in environments characterized by transient or externally influenced group formations. Rather than offering a static snapshot of cluster organization, the algorithm actively adjusts to connectivity and node behavior fluctuations. Moreover, the ‘’cluster synchronization framework‘’ is inherently scalable, making it highly effective for large-scale distributed systems, significantly reducing latency and enhancing fault tolerance by decentralizing control and minimizing reliance on centralized data aggregation.

To validate our methodology, we conducted experiments on both synthetic and real-world datasets, demonstrating its robustness and adaptability across diverse conditions. A comparative analysis with baseline methods demonstrates the superior effectiveness of our approach in achieving accurate and scalable clustering.

### Related works

When confronted with datasets too large for a single machine or spread across diverse locations, distributed clustering presents a notably effective strategy. The paper [4] comprehensively reviews various implementations of k-means clustering algorithms for handling large-scale datasets, providing a detailed comparative analysis of these implementations, highlighting their optimizations for enhanced performance on extensive datasets. Additionally, the article compiles an inclusive list of datasets used for benchmarking to ensure a rigorous evaluation, enabling a thorough assessment of each algorithm’s effectiveness under diverse data conditions.

The paper [5] presents innovative algorithms for distributed clustering tailored to k-means and k-median objectives. These methods address the challenge of communication complexity in distributed systems by leveraging coreset construction being a technique that compresses the data into a more miniature representation, although maintaining clustering accuracy enabling efficient computation across diverse networks. Experimental evaluations on large datasets reveal that these approaches outperform existing coreset-based distributed clustering methods. The authors suggest both theoretical proofs and experimental findings based on synthetic and real-world data to substantiate the efficacy of the methods. While reducing data transmission, this method incurs high computational overhead. Moreover, it lacks built-in privacy mechanisms for secure distributed clustering.

The study proposed in [6] introduces a unified framework for gradient-based distributed data clustering, solving optimization problems with several objectives presenting a family of algorithms tailored for clustering in distributed environments. This framework is designed to handle large-scale datasets distributed across multiple nodes, emphasizing improved communication efficiency and clustering performance in decentralized networks. The experimental results demonstrate the effectiveness of the approach across various settings, particularly in scenarios where a small number of agents manage large datasets. This approach is designed for scenarios with small agent groups and large local datasets, making it inefficient for systems with a large number of agents and limited local data per node.

In [7], a distributed k-means algorithm is introduced to mitigate node communication problems. The standout feature is quantized messages for more efficient data transmission to neighboring nodes. Note that, unlike the method described here, the algorithm presented further in the present article exploits compressed sensing. While this approach in [7] minimizes data transmission, it lacks robustness in highly dynamic environments with rapid cluster structure evolution. The scenario examined in [8], specifically the analysis of small datasets across a large number of nodes bears a partial resemblance to the context investigated in this paper. The method employs consensus algorithms. Max consensus is used for cluster initialization through leader elections. Finite-time average consensus enables agents to compute cluster centroids using only neighbor communications in a distributed manner. The method has high latency in achieving consensus and does not efficiently adapt to time-sensitive scenarios. The article [9] presents a refined penalized clustering model to analyze large-scale fused data using a nature-inspired optimization algorithm to improve the clustering performance. The study does not explicitly address real-time clustering in decentralized systems and lacks attention to privacy.

The cluster consensus problem consists of two key components: clustering algorithms and control mechanisms. As demonstrated in [10–13], early research primarily focused on centralized clustering methods such as genetic algorithms, bipartite matching, and k-means clustering. These approaches rely heavily on global information, which can pose challenges when access to comprehensive data is limited or unreliable.

Conversely, research in [14–17] has emphasized the development of distributed control laws to achieve cluster consensus in multi-agent systems across diverse scenarios, including swarm robotics, higher-order systems, and networks with antagonistic interactions. However, these approaches often face challenges regarding the controller complexity and computational overhead, as exemplified by the Riccati equation-based solutions in [15,16]. These studies often overlook the integration of dynamic clustering methods, relying instead on fixed or time-varying communication topologies [14–16] . This can lead to reduced adaptability and outdated clustering results, as these methods fail to account for real-time changes in system states or datasets. Limited coupling strengths or network connectivity further restrict their applicability. Specifically, the method in [14] relies on complex controllers, leading to high communication overhead and limiting scalability in large networks. The approach [16] introduces a significant computational burden and lacks inherent privacy preservation mechanisms and is unsuited for rapidly changing topologies and suffers from high communication operating costs. The performance of the approach discussed in [17] is contingent upon predefined clustering, and it does not prioritize fast consensus building. While more straightforward distributed control strategies, such as those presented in [18], offer more straightforward implementation, they are frequently modified to specific scenarios like containment control with fixed communication topologies that can constrict their flexibility in real-world applications.

Another issue connected to the problematics discussed is network systems entailing many interacting nodes communally functioning in an unknown environment. These types of systems are prevalent in numerous practical applications, garnering a significant interest within the research community. Examples include swarm robotics, distributed sensor networks, and electrical power grids, as illustrated in [19–21].

However, the algorithms mentioned are computationally expensive for high-dimensional data. In practice, data often have a sparse structure due to compact clustering of node states, and in such cases, a new paradigm like Compressed Sensing (CS) can be useful. CS [22,23] is an innovative and rapidly growing research area that has attracted significant interest across multiple disciplines, including electrical engineering, applied mathematics, statistics, and computer science. CS provides a framework for simultaneously sensing and compressing finite-dimensional vectors through linear dimensionality reduction. Its critical insight of CS is that high-dimensional sparse signals can be accurately reconstructed from a limited number of measurements using computationally efficient algorithms. However, the authors do not mention published works explicitly addressing the application of the CS theory combined with deep learning methods for distributed clustering, which have been used extensively in various domains, integrating these methodologies for distributed clustering remains less explored in the literature.

Recent advancements in CS have yielded cutting-edge architectures and methodologies that address challenges such as overfitting, computational efficiency, and scalability. CombNet [24] exemplifies this innovation by employing deeper neural architectures paired with smaller convolutional kernels, effectively minimizing overfitting while improving performance. DeepInverse [25] introduces a transformative approach by replacing fully convolutional layers with a transposed measurement matrix for initial signal reconstruction. This adaptation reduces the model’s complexity, allowing for efficient processing of long signals without block division. The method achieves faster computations and enhanced reconstruction quality, particularly at low compression ratios. Expanding upon this, DeepCodec [26] incorporates a learned sampling mechanism customized to the training dataset, improving reconstruction accuracy and reducing computational overhead. Similarly, ConvCSNet [27] adopts a learnable measurement matrix but utilizes linear layers for compression. Its dual-branch CNN-based architecture enables it to process full-sized signals effectively, like DeepInverse. Residual Networks (ResNet), which mitigate vanishing gradient issues in deep learning, have also been integrated into CS. For instance, the Deep Residual Reconstruction Network [28] combines adaptive sampling techniques with ResNet blocks to optimize reconstruction performance. Generative Adversarial Networks (GANs) have also significantly evolved in CS applications. For instance, [29] augments the ReconNet framework by incorporating a discriminator that distinguishes between real and generated data. This setup enables the generator to map compressed measurements directly to reconstructed outputs, achieving efficient and accurate signal recovery. In MRI reconstruction, [30] leverages GANs with ResNet-based generators and CNN-based discriminators. Despite the inherent challenges in GAN stability, [31] simplifies this process by focusing on detail completion, leading to faster convergence and streamlined models.

### Main advantages and contributions

The proposed method addresses several key limitations of previously published works by focusing on communication efficiency, privacy preservation, and fast consensus in decentralized clustering.

**Advantages:**

- *Communication Efficiency:* By leveraging compressed sensing, data exchange is minimized while maintaining clustering accuracy, enhancing scalability for large distributed systems.
- *Privacy Preservation:* Conventional methods often involve sharing raw or partially processed data. In contrast, the proposed approach shares only aggregated data, applying randomized transformations to ensure data security.
- *Fast Consensus:* Utilizing an accelerated consensus algorithm enables quicker stabilization of clustering assignments, making the method well-suited for dynamic environments.
- *Scalability:* The approach is optimized for networks with numerous agents and limited local data per node, overcoming the constraints of existing methods that assume access to extensive datasets.

**Contributions:**

- This work introduces a novel decentralized clustering framework that integrates compressed sensing with an accelerated consensus protocol to gather and process compressed states across a network efficiently. By reducing dimensionality and employing consensus mechanisms for distributed data aggregation, the system enables nodes to estimate the global cluster structure while minimizing data transmission collaboratively. Each node independently generates compact representations that encapsulate clustering structures, ensuring consistency across the network. A pre-trained neural network reconstructs the overall cluster organization, enabling efficient large-scale data processing. Unlike traditional distributed clustering methods focusing solely on fixed or time-varying network topologies, the approach dynamically adapts to evolving states in real-time, requiring minimal computational resources.
- The integration of compressed sensing with a consensus mechanism facilitates decentralized grouping. The offline phase involves training a neural network on an artificial dataset to define expected cluster configurations. During the online phase, nodes generate compact representations of the clustering structure, which the trained model processes to reconstruct the global state. By offloading resource-intensive tasks such as dataset creation and neural network training to the offline phase, our approach ensures an efficient and adaptable clustering solution for dynamic network environments.

## Problem statement

### Motivation

For coalitional control to be effective in multi-agent systems, the accurate and efficient reconstruction of cluster structures following coalition formation is essential [32]. Although coalition control is acknowledged as a strong alternative to centralized and fully decentralized approaches, the ability to efficiently identify and maintain coalition structures in dynamic environments remains a significant obstacle to its practical deployment.

Two opposite paradigms are commonly recognized in traditional multi-agent control: centralized methods, such as model predictive control and optimal control, which necessitate solving complex, large-scale optimization problems resulting in substantial computational and communication overhead, rendering them impractical for large systems; and fully decentralized methods, which eliminate the need for global coordination but often fail to establish structured coalition formation, leading to suboptimal performance in cooperative tasks.

To address the limitations of current methods, this paper introduces a communication-efficient decentralized clustering method, enabling accurate and scalable reconstruction of cluster structures in multi-agent systems. In contrast to conventional clustering methods, which depend on global information or iterative optimization, our approach facilitates efficient coalition membership inference through local interactions, thereby minimizing communication overhead while preserving high cluster structure recovery accuracy.

Accurately reconstructing cluster structures is crucial for coalitional control, as it enables scalable decision-making by reducing computational complexity and facilitating real-time coalition-based control. Furthermore, it enhances communication efficiency by allowing agents to reconstruct coalition structures through localized interactions, minimizing unnecessary data exchange. Finally, it provides robust adaptation in dynamic environments, enabling agents to quickly and accurately reassess their coalition membership, thereby improving system resilience and coordination efficiency.

The proposed method is particularly beneficial for applications such as autonomous vehicle fleets, intelligent transportation systems, distributed sensor networks, and energy grid management, where maintaining coalitional structures is crucial despite dynamic agent behavior and communication constraints. For instance, in multi-agent robotic navigation within unknown environments, robots must adaptively form clusters to maneuver around obstacles. Traditional decentralized clustering methods would necessitate continuous updates, resulting in high communication costs and instability. However, this approach assumes that cluster evolution occurs at a slower timescale than local control actions and employs an accelerated consensus algorithm, ensuring stable clustering decisions during critical operations. This enables agents to coordinate efficiently without excessive communication overhead. By creating a decentralized clustering mechanism that reconstructs coalition structures with minimal communication overhead, this research aims to improve the scalability, adaptability, and efficiency of multi-agent control systems, fully leveraging coalitional control in real-world scenarios.

### System description

Consider a multi-agent system consisting of a set of agents 𝒩={1,…,n}. Each agent i∈𝒩 has dynamics, which is modeled by the following state-space equation:

$${𝐱}_{i}(t+1)=f_{i}({𝐱}_{i}(t),{𝐮}_{i}(t),{𝐰}_{i}(t+1)),$$ (1)

where ${𝐱}_{i}(t)\in {\mathbb{R}}^{d}$ is the state vector, ${𝐮}_{i}(t)\in {\mathbb{R}}^{q}$ is the control input, ${𝐰}_{i}(t)\in {\mathbb{R}}^{p}$ is the external disturbance, and $f_{i}:{\mathbb{R}}^{d}\times {\mathbb{R}}^{q}\times {\mathbb{R}}^{p}\to {\mathbb{R}}^{d}$ is a possibly nonlinear function describing the dynamics of agent *i*.

By aggregating the local variables, we define global vectors:


$$\bar{x}(t)=col({𝐱}_{1}(t),\ldots ,{𝐱}_{n}(t))\in {\mathbb{R}}^{nd},$$

$$\bar{u}(t)=col({𝐮}_{1}(t),\ldots ,{𝐮}_{n}(t))\in {\mathbb{R}}^{nq},$$

$$\bar{w}(t)=col({𝐰}_{1}(t),\ldots ,{𝐰}_{n}(t))\in {\mathbb{R}}^{np}.$$

The overall system dynamics can then be expressed as:

$$\bar{x}(t+1)=F(\bar{x}(t),\bar{u}(t),\bar{w}(t+1)),$$ (2)

where $F:{\mathbb{R}}^{nd}\times {\mathbb{R}}^{nq}\times {\mathbb{R}}^{np}\to {\mathbb{R}}^{nd}$ is the aggregated mapping function. This model type is commonly used for controlling large-scale systems, such as vehicle platoons [33] and traffic networks [34].

#### Network layer.

We assume that agents communicate with each other through a network. In many multi-agent systems, the structure of the communication network is not static, but rather evolves over time. This evolution can be due to a variety of factors, such as mobility of agents, changing network conditions, or dynamic interactions between agents. In such systems, time-varying graphs are used to exhibit the time-dependent nature of communication. Formally, such a model can be represented as a sequence of graphs $\{G(t){\}}_{t\in T}$, where G(t)=(V,E(t)) is the current graph at time *t*, with

- *V* is the set of vertices (agents), assumed to remain constant over time.
- E(t)⊆V×V is the set of edges at time *t*, which can change from one time step to another.

Each edge $(v_{i},v_{j})\in E_{t}$, i,j∈𝒩, signifies that agents $v_{i}$ and $v_{j}$ can communicate at time *t*, and these edges may be time-dependent, reflecting the presence or absence of communication links at specific time intervals. We assume that the communication is directed, meaning $\left(v_{i},v_{j})\in E(t\right)$ does not imply $\left(v_{j},v_{i})\in E(t\right)$.

**Assumption 1** (Expected strong connectivity) *Let B(t) denote the adjacency matrix of the directed communication graph G(t). We assume that the expected graph ${𝒢}_{\text{avg}}$, defined by the expected adjacency matrix 𝔼[B(t)], is strongly connected. That is, the graph induced by 𝔼[B(t)] contains a directed path between every pair of nodes, ensuring that information can propagate globally in expectation.*

The assumption implies that, although individual realizations *G*(*t*) may be disconnected at any given time, the network is connected on average, allowing for global information flow in the long run. This strong connectivity guarantees that information can propagate in both directions across the network, ensuring the effectiveness of distributed algorithms and the convergence of aggregation procedures.

The **in(incoming)-neighbor set** of an agent $v_{i}$ at time *t*, denoted by ${𝒩}_{i}(t)$, is the set of agents to which $v_{i}$ is directly connected at time *t*. Mathematically:


$${𝒩}_{i}(t)=\{v_{j}\in V|(v_{j},v_{i})\in E(t)\}.$$

The weighted in-degree of node *i* is ${deg}_{i}^{+}(B(t))=\sum_{j=1}^{n}b_{i,j}(t)$, and the Laplacian matrix is ℒ(B(t))=𝒟(B(t))−B(t), where *B*(*t*) corresponds to the weighted adjacency matrix at time *t*, $𝒟(B(t))=diag({deg}_{1}^{+}(B(t)),\ldots ,{deg}_{n}^{+}(B(t)))$.

#### Structural layer.

Agents in large-scale systems commonly group into coalitions or clusters to simplify operations and increase scalability. We define a coalitional structure $𝒞(t)=\{C_{1}(t),\ldots ,C_{n_{c}}(t)\}$ as a partition of the global set 𝒩 into $n_{c}\in [1,n]$ coalitions (or clusters), where each coalition *C*_*l*_(*t*), $l\in \{1,\ldots ,n_{c}\}$, is suggested to include states sharing similar characteristics.

The coalitions must satisfy the conditions: ${𝒞}_{l}(t)\neq \emptyset$ for all *l*, ${⋃}_{l=1}^{n_{c}}{𝒞}_{l}(t)=𝒩$, and ${𝒞}_{l}(t)\cap {𝒞}_{\bar{l}}(t)=\emptyset$ for $l\neq \bar{l}$. Each coalition ${𝒞}_{l}(t)$ is considered as a subsystem with aggregated dynamics:

$${𝐱}_{{𝒞}_{l}(t)}(t+1)=F({𝐱}_{{𝒞}_{l}(t)}(t),{𝐮}_{{𝒞}_{l}(t)}(t),{𝐰}_{{𝒞}_{l}(t)}(t+1),$$ (3)

where ${𝐱}_{{𝒞}_{l}(t)}(t)=col({𝐱}_{i}(t){)}_{i\in {𝒞}_{l}(t)}$, ${𝐮}_{{𝒞}_{l}(t)}(t)=col({𝐮}_{i}(t){)}_{i\in {𝒞}_{l}(t)}$, and ${𝐰}_{{𝒞}_{l}(t)}(t)=col({𝐰}_{i}(t){)}_{i\in {𝒞}_{l}(t)}$.

While conventional approaches assume a pre-defined coalitional structure with individual coalition control input synthesis, self-organizing systems necessitate agents to dynamically determine their cluster membership based on state vectors and neighbor measurements.

**Assumption 2. (Cluster stability during clustering process).** *Once the clustering algorithm groups agents into clusters, the assignment of agents to clusters remains fixed throughout the process. There are no dynamic changes, such as a cluster merging, splitting, or shifting significantly once the initial groupings are established.*

The rationale behind Assumption 2 is that state relationships are assumed to be stable throughout the clustering process. As a result, clustering processes are handled separately, and time indices are excluded for simplification. These processes may occur over longer durations, with timescales ranging from minutes to seasonal periods, such as indicated in [32].

**Remark 1.** *Assumption 2 establishes a constraint of fixed cluster assignments during the clustering process. Although this constraint might not apply to highly dynamic decentralized networks, the analysis focuses on scenarios where cluster evolution occurs slower than local control actions. For example, in mobile robot navigation within an unknown environment, clusters dynamically form during obstacle avoidance maneuvers but remain stable between them. Re-clustering events, while possible, are infrequent compared to local control updates, rendering the assumption practical for applicable scenarios.*

### Objective

The objective is to discover the coalitional structure through communication-efficient local agent interactions. The central challenge in distributed clustering lies in developing algorithms that can effectively partition agents into meaningful clusters while minimizing centralized control and inter-agent communication overhead. Therefore, the problem encompasses the following subtasks:

- **Coalition Formation:** Based on a defined clustering criterion, agents are required to decide which states belong to a coalition. Each agent must perform this assignment locally, without knowledge of all states. The aim is to achieve an optimal partition that minimizes a clustering objective, which generally measures similarity within coalitions and dissimilarity between them.
- **Communication Efficiency:** Due to the cost or limitations of inter-agent communication, the goal is to minimize information exchange during clustering. Ideally, the data transmitted between nodes should scale sub-linearly with the number of agents or data points. The communication overhead can be embodied as:
  $${𝒞}_{\text{comm}}=\sum_{i=1}^{n}|{ℳ}_{i}|,$$
- where ${ℳ}_{i}$ represents the set of messages exchanged between agent *i* and its neighbors. The message size is defined as the number of packets exchanged between an agent and its neighbors. We assume that each packet carries one entry of a vector. The purpose is to minimize ${𝒞}_{\text{comm}}$ while ensuring convergence to an optimal or near-optimal clustering solution.

## Decentralized clustering through CS and ML

Effective decentralized clustering, with communication efficiency, requires the optimization of a particular objective function alongside the reduction of communication overhead. A distributed optimization of an objective, akin to that used in the classical k-means algorithm, is feasible if each node has access to the relevant centroid. Since centroid calculation requires complete state information from all agents in a cluster, two common strategies are typically employed. One approach involves centralizing all data, which improves accuracy but reduces communication efficiency. Alternatively, centroids can be computed in a distributed manner to minimize data exchange. This paper proposes a method that applies compressed sensing to compress local agent states. The compressed data is then processed through a consensus mechanism to estimate the global state, allowing each node to reconstruct the centroids independently using a predictive model.

### Decentralized compressed data aggregation

The solution presented comprises two primary components: the sensing and communication stages.

**Sensing stage.** Multi-agent systems involve deploying numerous agents across a spatial region, for example, in sensor networks or IoT deployments. These agents independently collect local data, which is frequently sparse or compressible using transforms like wavelets or Fourier. Due to their independent operation, agents might not have access to the entire dataset or all data at once. The data acquisition process is sparse, recording only notable events or changes.

Given a compressible signal $𝐱\in {\mathbb{R}}^{d}$, it can be represented sparsely as $𝐳\in {\mathbb{R}}^{d}$ (a vector with many zero entries) using the sparsifying basis $\Psi \in {\mathbb{R}}^{d\times d}$:

𝐳=Ψ𝐱. (4)

Agent state representations often include features like positions, sensor readings, or other parameters, which are high-dimensional, continuous, and dense. These states are commonly mapped onto a two-dimensional grid to improve processing, storage, or communication efficiency. This transformation frequently yields a sparse representation. Each agent’s state, such as its position or value of interest, is assigned to a grid cell based on its coordinates or features. For instance, a position (v,w) can be mapped to the grid cell corresponding to the nearest grid coordinates. When the state is a multi-dimensional vector, dimensionality reduction techniques, like PCA or t-SNE, can project the state onto a 2D plane before grid mapping. Typically, this mapping is non-linear and not considered a sparsifying basis Ψ. However, our method bypasses the reconstruction step, enabling direct utilization of the sparse signal from the mapping, effectively treating Ψ as an identity matrix.

To represent agent *i*’s position on a 2D grid, we define a sparse vector ${\tilde{𝐱}}_{i}\in {\mathbb{R}}^{g_{s}}$, containing zeros and a single one, based on its state ${𝐱}_{i}$. Here, *g*_*s*_ denotes the total number of grid cells. From these vectors, we construct a common normalized vector $\tilde{\text{X}}=\frac{1}{\sqrt{n}}\sum_{i}{\tilde{𝐱}}_{i}$, where $|\tilde{\text{X}}|=1$.

The coalitional structure allows for a sparse encoding $\tilde{\text{Z}}$ of $\tilde{\text{X}}$. For each coalition ${𝒞}_{l}$, where $l=1,\ldots ,n_{c}$, we define a one-hot vector ${\tilde{\mu }}_{l}\in {\mathbb{R}}^{g_{s}}$ that marks the grid position corresponding to the centroid $\mu_{l}$ of coalition ${𝒞}_{l}$. We also define the cumulative vector of agents in coalition ${𝒞}_{l}$ as: ${\tilde{\text{C}}}_{l}=\sum_{i\in {𝒞}_{l}}{\tilde{𝐱}}_{i}$.

Finally, we introduce the transformation $\Psi_{\varepsilon }$ given by:


$$\Psi_{\varepsilon }=\varepsilon I_{g_{s}\times g_{s}}+\sqrt{\frac{n(1-\varepsilon^{2})}{\sum_{l=1}^{n_{c}}|{𝒞}_{l}{|}^{2}}}\sum_{l=1}^{n_{c}}{\tilde{\mu }}_{l}{\tilde{\text{C}}}_{l}^{T},$$

where $\left|{𝒞}_{l}\right|$ denotes the cardinality of the set ${𝒞}_{l}$, i.e., the number of agents in coalition *l*, ε is an arbitrarily small positive parameter.

Applying this transformation to the common vector yields the sparse representation:


$$\tilde{\text{Z}}=\Psi_{\varepsilon }\tilde{\text{X}}.$$

The resulting vector $\tilde{\text{Z}}$ approximates the common sparse centroid vector:


$$\bar{\text{Z}}=\frac{1}{\sqrt{\sum_{l=1}^{n_{c}}|{𝒞}_{l}{|}^{2}}}\sum_{l=1}^{n_{c}}{\tilde{\mu }}_{l}|{𝒞}_{l}|,$$

which has exactly *n*_*c*_ non-zero components corresponding to the coalition centroids.

The approximation error between $\tilde{\text{Z}}$ and $\bar{\text{Z}}$ is bounded in the $\ell_{1}$ norm as:


$$\|\bar{\text{Z}}-\tilde{\text{Z}}{\|}_{1}\leq \varepsilon \sqrt{n}.$$

Utilizing compressed sensing, each agent reduces its state representation by applying random projections, thus minimizing data transmission or storage. The measurements are obtained by multiplying the original data with a sensing matrix $\Phi \in {\mathbb{R}}^{m\times g_{s}}$, where $m\ll g_{s}$, resulting in a compressed data version, ${𝐲}_{i}=\Phi {\tilde{x}}_{i}$. This technique allows for acquiring fewer data points than the original dataset:

$$\tilde{\text{Y}}=\Phi \tilde{\text{X}}=\frac{1}{\sqrt{n}}\sum_{i=1}^{n}\Phi {\tilde{x}}_{i}=\sqrt{n}\frac{1}{n}\sum_{i=1}^{n}{𝐲}_{i}$$ (5)

where ${𝐲}_{i}$ is a column of Φ corresponding to a nonzero entry of ${\tilde{𝐱}}_{i}$.

**Communication stage.** In traditional systems, each agent would send raw data to a central server or another aggregation point. However, in the multi-agent systems studied here, only the compressed measurements (random projections) are transmitted across the network that drastically reduces the bandwidth required for communication.

To facilitate independent cluster assignments, each agent must aggregate the compressed measurements from all agents, for example, by combining them into a single unified compressed representation. This aggregation can be achieved through various methods, such as linear combinations: $𝐲=\frac{1}{n}\sum_{i=1}^{n}{𝐲}_{i}.$

Consensus-based algorithms offer a decentralized solution for aggregation. Consensus involves agents sharing and combining information to reach a widespread agreement, ensuring system consistency while maintaining decentralization. In the context of compressed measurements, consensus-based aggregation allows agents to compute a shared representation, preserving individual data privacy. Typically, this is achieved through iterative algorithms where agents update their estimates based on neighbor measurements. Common consensus protocols include the Gossip algorithm and Weighted Average [35], designed for convergence to a common value. We employ an iterative scheme, starting with initial vectors ${𝐲}_{i}$ and converge to the target vector y as iterations increase. However, we restrict the process to a finite number of iterations due to practical limitations. Consequently, y is approximated with an accuracy level of $\epsilon_{2}$.

#### Reliable cluster structure recovery under incomplete and inaccurate measurements.

The following convex optimization problem is considered, where the objective is to find the cluster structure with the smallest $\ell_{1}$-norm consistent with the observed data $\tilde{\text{Y}}$. The problem is formulated as:

$$\min\|\tilde{\text{Z}}{\|}_{1}\;\;\;\text{subject to }\;\;\;\|A\tilde{\text{Z}}-\tilde{\text{Y}}{\|}_{2}\leq \epsilon_{1},$$ (6)

where the matrix *A* is defined as $A=\Phi \Psi_{\varepsilon }$, $\epsilon_{1}>0$ is the tolerance parameter account for measurement noise or inaccuracies in the observed data $\tilde{\text{Y}}$.

The uniform uncertainty principle asserts that the measurement matrix $A\in {\mathbb{R}}^{m\times g_{s}}$ satisfies a *restricted isometry property* (RIP) [36]. Specifically, there exists a constant δ(s), known as the *s-restricted isometry constant*, such that for all vectors Z with sparsity at most *s*, the following inequality holds:

$$(1-\delta (s))\|\text{Z}{\|}_{2}\leq \|A\text{Z}{\|}_{2}\leq (1+\delta (s))\|\text{Z}{\|}_{2}.$$ (7)

The smallest value of δ(s) that satisfies inequality (7) is accepted as the assessment of δ(s).

Let ${\tilde{\text{Z}}}^{⋆}$ denote the solution of the convex optimization problem (6). According to Theorem 2 from [22], the deviation between ${\tilde{\text{Z}}}^{⋆}$ and the actual cluster structure $\bar{\text{Z}}$ is bounded as follows:

$$\|{\tilde{\text{Z}}}^{⋆}-\bar{\text{Z}}\|\leq c_{1}\epsilon_{1}+c_{2}\epsilon_{2},$$ (8)

providing that the measurement matrix *A* satisfies the RIP condition (7) with $\delta (3n_{c})+3\delta (4n_{c})<2$. The term $\epsilon_{2}$ is chosen such that


$$\epsilon_{2}\geq \frac{\|\bar{\text{Z}}-\tilde{\text{Z}}{\|}_{1}}{\sqrt{n_{c}}}=\varepsilon \sqrt{\frac{n}{n_{c}}}.$$

In practical scenarios, the constants *c*_1_ and *c*_2_ in (8) remain within acceptable ranges for $\delta (4n_{c})$; for instance, if $\delta (4n_{c})=\frac{1}{5}$, then $c_{1}\approx 12.04$ and $c_{2}\approx 8.77$ [22].

The objective, in line with the restricted isometry property, is to design a sensing matrix *A* with approximately orthogonal columns for any chosen subset.

#### Design of the measurement matrix.

A direct construction of the measurement matrix Φ, such that $A=\Phi \Psi_{\varepsilon }$ satisfies the RIP condition, would require verifying the inequality (7) for all (gsnc)=gs!nc!(gs−nc)! possible combinations of positions for the *n*_*c*_ nonzero entries in the vector $\tilde{Z}\in {\mathbb{R}}^{g_{s}}$. However, it has been shown that the RIP property can be fulfilled with a high probability by simply choosing Φ as a random matrix using randomization of the observation process [37]. In this setting, the measurement vector $\tilde{\text{Y}}$ consists of *m* linear combinations of the components of $\tilde{\text{X}}$, with the weights in each combination selected randomly.

A random matrix $\Phi \in {\mathbb{R}}^{m\times g_{s}}$, whose entries are independently and identically distributed (i.i.d.) as $\Phi [i,j]\sim 𝒩\left(0,\frac{1}{m}\right)$, exhibits two important and useful properties [22]:

- If 0<δ(m)<1 and
  $$m\geq c_{3}\;n_{c}\;\log\left(\frac{g_{s}}{n_{c}}\right),$$ (9)
- then the matrix Φ satisfies the RIP condition with probability at least $1-2e^{-c_{4}m}$, where *c*_3_ and *c*_4_ are positive constants that depend only on δ. This implies that any *n*_*c*_-sparse or compressible vector of length *g*_*s*_ can be recovered with high probability from only $m\ll g_{s}$ random measurements.
- The matrix Φ is **universal** in the sense that it preserves the essential information of any *n*_*c*_-sparse signal during the dimensionality reduction from $\tilde{X}\in {\mathbb{R}}^{g_{s}}$ to $𝐲\in {\mathbb{R}}^{m}$. Furthermore, for any choice of orthonormal basis $\Psi_{\varepsilon }$, the product $A=\Phi \Psi_{\varepsilon }$ is itself a random matrix with i.i.d. Gaussian entries and thus inherits the RIP property with the same high probability.

As shown in [38], the condition (9) can be derived directly from the well-known Johnson–Lindenstrauss lemma [39], which states that any set of *n* points in a *d*-dimensional Euclidean space can be embedded into an *m*-dimensional Euclidean space, where m~logn and is independent of *d*, such that all pairwise distances are approximately preserved.

In the context of the random matrix Φ, the universality of its behavior and specific constraints for selecting the constants *c*_3_ and *c*_4_ are also discussed in [37,38]. These constants satisfy the following inequality:


$$c_{4}\leq c_{0}(\delta /2)-c_{3}\left(1+\frac{1+\log(12/\delta )}{\log(g_{s}/n_{c})}\right),\;\text{where}\;c_{0}(\delta /2)=\frac{\delta^{2}}{16}-\frac{\delta^{3}}{48}.$$

#### RIP-preserving transformation.

The success of sparse recovery via convex optimization, as expressed in the bound (8), relies on the measurement matrix $A=\Phi \Psi_{\varepsilon }$ satisfying the restricted isometry property with a sufficiently small constant δ(s) for sparsity level $s\sim n_{c}$. While the random matrix $\Phi \in {\mathbb{R}}^{m\times g_{s}}$ satisfies the RIP with high probability under standard conditions as discussed above, it is crucial to ensure that the composite matrix *A* inherits this property after applying the structured transformation $\Psi_{\varepsilon }$.

To ensure that the coalition-based transformation $\Psi_{\varepsilon }$ remains well-behaved and does not introduce excessive imbalance, we impose a mild regularity condition on the coalition structure. Specifically, we require that the coalition sizes are uniformly bounded around their average value. This prevents extreme disparities between large and small coalitions, which could otherwise affect the stability of the representation and the RIP property of the resulting measurement matrix.

**Assumption 3. (Approximate balance of coalition sizes).** *The coalition structure is approximately balanced if there exists a constant κ≥1 such that for all $l=1,\ldots ,n_{c}$, the size of each coalition satisfies:*


$$\frac{n}{\kappa n_{c}}\leq |{𝒞}_{l}|\leq \kappa \cdot \frac{n}{n_{c}}.$$

That is, each coalition contains a number of agents that deviates from the average $\frac{n}{n_{c}}$ by at most a factor of κ.

**Remark 2. (Interpreting the balance parameter κ).** *The parameter κ determines how far individual coalition sizes are allowed to deviate from the average. When κ=1, all coalitions are perfectly balanced and have equal size. For κ>1, moderate deviations are allowed: the larger κ, the more heterogeneous the coalition structure may be. This formulation ensures that coalitions remain well-proportioned while still allowing flexibility. Importantly, the condition scales naturally with both the number of agents n and the number of coalitions *n**_*c*_*, making it suitable for large-scale systems.*

The following proposition provides an explicit upper bound that plays a central role in ensuring that the measurement matrix $A=\Phi \Psi_{\varepsilon }$ preserves the restricted isometry property. The result relies on the structural assumptions introduced above, particularly the approximate balance of coalition sizes and the orthogonality of the centroid indicators.

**Proposition 1. (IP-preserving bound).** *Under Assumption 3, the transformation matrix $\Psi_{\varepsilon }$ satisfies the operator norm bound*


$$\left\|\Psi_{\varepsilon }-\varepsilon I_{g_{s}\times g_{s}}\right\|\leq \delta_{\varepsilon },\;\text{where}\;\delta_{\varepsilon }=\kappa \cdot \sqrt{\frac{(1-\varepsilon^{2})n}{n_{c}}}.$$

*If $\delta_{\varepsilon }$ is sufficiently small, then the matrix $A=\Phi \Psi_{\varepsilon }$ satisfies the RIP condition:*


$$\delta (3n_{c})+3\delta (4n_{c})<2.$$

**Proof 1.** *Let us write:*


$$\Psi_{\varepsilon }=\varepsilon I_{g_{s}\times g_{s}}+\Delta ,\;\Delta :=\sqrt{\frac{n(1-\varepsilon^{2})}{\sum_{l=1}^{n_{c}}|{𝒞}_{l}{|}^{2}}}\sum_{l=1}^{n_{c}}{\tilde{\mu }}_{l}{\tilde{\text{C}}}_{l}^{T}.$$

*Since the vectors ${\tilde{\mu }}_{l}$ have disjoint support, each matrix ${\tilde{\mu }}_{l}{\tilde{\text{C}}}_{l}^{T}$ has nonzero entries only in a single row (determined by the support of ${\tilde{\mu }}_{l}$, i.e. the centroid vectors have non-overlapping support, which we express via orthogonality: ${\tilde{\mu }}_{l}^{T}{\tilde{\mu }}_{l^{\prime }}=0,\forall l\neq l^{\prime }.$). As a result, the matrices ${\tilde{\mu }}_{l}{\tilde{\text{C}}}_{l}^{T}$ are orthogonal in row space, and, using the definition of the spectral norm, we obtain:*


$$\left\|\sum_{l=1}^{n_{c}}{\tilde{\mu }}_{l}{\tilde{\text{C}}}_{l}^{T}\right\|=\max_{1\leq l\leq n_{c}}\|{\tilde{\text{C}}}_{l}{\|}_{2}.$$

*Each ${\tilde{\text{C}}}_{l}$ is a sum of one-hot vectors, so its entries are non-negative integers counting the number of agents occupying each grid cell. Hence:*


$$\|{\tilde{\text{C}}}_{l}{\|}_{2}\leq |{𝒞}_{l}|\leq \kappa \cdot \frac{n}{n_{c}}.$$

*Next, we apply the Cauchy–Schwarz inequality and estimate the normalization factor:*


$$\sum_{l=1}^{n_{c}}|{𝒞}_{l}{|}^{2}\geq \frac{1}{n_{c}}{\left(\sum_{l=1}^{n_{c}}|{𝒞}_{l}|\right)}^{2}=\frac{n^{2}}{n_{c}}\;\Rightarrow \;\sqrt{\frac{n(1-\varepsilon^{2})}{\sum_{l=1}^{n_{c}}|{𝒞}_{l}{|}^{2}}}\leq \sqrt{\frac{(1-\varepsilon^{2})n_{c}}{n}}.$$

*By combining both bounds*


$$\|\Delta \|\leq \sqrt{\frac{(1-\varepsilon^{2})n_{c}}{n}}\cdot \kappa \cdot \frac{n}{n_{c}}=\kappa \cdot \sqrt{\frac{(1-\varepsilon^{2})n}{n_{c}}},$$

*we get*


$$\left\|\Psi_{\varepsilon }-\varepsilon I\right\|=\|\Delta \|\leq \kappa \cdot \sqrt{\frac{(1-\varepsilon^{2})n}{n_{c}}}.$$

*This completes the proof.*

### Optimization-based consensus

Standard consensus algorithms often suffer from slow convergence, requiring numerous iterations to reach a solution. In real-world applications, faster convergence is critical for system efficiency, responsiveness, and overall performance. This is especially true for real-time or near-real-time decision-making systems like autonomous vehicles, robotics, financial trading, and sensor networks. In such scenarios, rapid consensus guarantees swift and accurate system decisions. For instance, autonomous vehicle fleets require quick consensus on traffic conditions or road hazards. To address this challenge, we propose an extension of the Accelerated Local Voting Protocol (Accelerated-LVP) from [40] to a vector-valued setting.

Consensus through optimization in multi-agent networks involves designing an optimization problem whose solution guides agents towards a shared decision. This approach enables decentralized consensus while preserving individual agent autonomy.

We aim to compute 𝐲 at each agent simultaneously using an iterative method. Let ${\hat{𝐲}}_{i}(k)$ represent the estimate of $\hat{𝐲}$ maintained by agent *i* at iteration *k*, where $k=0,1,\ldots ,n_{\text{lvp}}$. The initial estimate is set as ${\hat{𝐲}}_{i}(0)={𝐲}_{i}$, as defined in (5).

In this paper, to reach agreement on a common consensus vector $1_{m}⊗𝐲$, we propose minimizing a regularized consensus objective function, given by:

$$R(\bar{\hat{𝐲}})=\frac{\left(1+\lambda \right)}{2}\cdot {\bar{\hat{𝐲}}}^{T}({𝐈}_{m}⊗ℒ(B(t)))\bar{\hat{𝐲}},$$ (10)

where:

- $\bar{\hat{𝐲}}=[{\hat{𝐲}}_{1}^{⊤},\ldots ,{\hat{𝐲}}_{n}^{⊤}{]}^{⊤}$ denotes the vector of all compressed states.
- λ>0 is a regularization parameter.
- ⊗ denotes the Kronecker product, which is used to extend the Laplacian matrix to handle vector-valued states.
- ${𝐈}_{m}$ is the identity matrix of dimension m×m, corresponding to the fact that the state of each agent is a vector of dimension m.

This objective function minimizes the differences between neighboring agents ${\hat{y}}_{i}$ and ${\hat{y}}_{j}$ while incorporating a strong convexity regularizer.

The next step is to take partial derivatives with respect to *i*-th components ${\hat{𝐲}}_{i}$:

$${𝐠}_{i}=\frac{\partial R(\bar{\hat{𝐲}})}{\partial {\hat{𝐲}}_{i}}=(1+\lambda )\sum_{j\in {𝒩}_{i}(t)}b_{i,j}(t)\left({\hat{𝐲}}_{j}-{\hat{𝐲}}_{i}\right).$$ (11)

Finally, we need to determine the Lipschitz constant *L* and the strong convexity constant μ of the function to be optimized (10), as these values are essential for choosing the appropriate parameters:

- the **Lipschitz constant** *L* is:
  $$L=\frac{1+\lambda }{2}\cdot \lambda_{\max}(ℒ(B(t))),$$
- the **strong convexity constant** μ is:
  $$\mu =\frac{1+\lambda }{2}\cdot \lambda_{\min}(ℒ(B(t))),$$

where $\lambda_{\min}(ℒ(B(t)))$ refers to the smallest eigenvalue of the matrix ℒ(B(t)) and $\lambda_{\max}(ℒ(B(t)))$ refers to the largest eigenvalue of the matrix ℒ(B(t)).

Next, we incorporate our modifications into the Accelerated-LVP framework, resulting in Algorithm 1. This algorithm serves as the aggregation step within the main algorithm. Crucially, all agents simultaneously generate the algorithm’s output, namely the compressed measurement $\bar{𝐲}$, by processing only their local data and that of their immediate neighbors. After transmitting and aggregating these measurements, the subsequent stage involves reconstructing the centroids $\mu_{l}$, $l=1,\ldots ,n_{c}$.

**Algorithm 1 Accelerated-LVP.**

**Inputs:**

- Initial estimate ${\hat{𝐲}}_{i}(0)={𝐲}_{i}\in {\mathbb{R}}^{m}$ of agent *i*

- Step-size *h*>0 of gradient-like iteration

- Lipschitz constant *L*, strong convexity constant μ

- Regularization parameter λ>0

- Number of iterations $n_{lvp}$

**Output:** Compressed aggregated measurement ${\hat{𝐲}}_{i}\left(n_{lvp}\right)$

1: Pick η∈(0,μ), and set $H=h-\frac{h^{2}L}{2}$

2: Choose $\alpha_{x}\in (0,1)$ and set initial $\alpha_{0}=\alpha_{x}$

3: Copy initial estimate to auxiliary variable ${𝐯}_{0}={\hat{𝐲}}_{0}$

4: Define $\gamma_{0}>0$

5: **for** *k* = 1 to $n_{lvp}$ **do**

6:   Determine the local communication neighborhood ${𝒩}_{i}(k)$

7:   Request the compressed states ${\hat{𝐲}}_{j}(k)$ from the neighboring

agents

8:   Find $\alpha_{i}(k)$ by solving equation:


$$\alpha_{i}^{2}(k)=2(H-\frac{\alpha_{x}}{2})((1-\alpha_{i}(k))\gamma_{i}(k)+\alpha_{i}(k)(\mu -\eta ))$$

9:

10:   Update $\gamma_{i}(k)$:


$$\gamma_{i}(k+1)=(1-\alpha_{i}(k))\gamma_{i}(k)+\alpha_{i}(k)(\mu -\eta )$$

11:   Calculate auxiliary variable ${𝐬}_{i}(k)$:


$${𝐬}_{i}(k)=\frac{1}{\gamma_{i}(k)+\alpha_{i}(k)(\mu -\eta )}(\alpha_{i}(k)\gamma_{i}(k){𝐯}_{i}(k)+\gamma_{i}(k+1){\hat{𝐲}}_{i}(k))$$

12:   compute gradient ${𝐠}_{i}(k)$ at point ${𝐬}_{i}(k)$ using (11)

13:   Update the estimate:


$${\hat{𝐲}}_{i}(k+1)={𝐬}_{i}(k)-h{𝐠}_{i}(k)$$

14:

15:   Update auxiliary variable ${𝐯}_{i}(k)$:


$${𝐯}_{i}(k+1)=\frac{1}{\gamma_{i}(k)}[(1-\alpha_{i}(k))\gamma_{i}(k)v_{i}(k)+\alpha_{i}(k)(\mu -\eta ){𝐬}_{i}(k)-\alpha_{i}(k){𝐠}_{i}(k)]$$

16: **end for**

Accelerated-LVP introduces an additional modeling layer that affects how well the aggregated state of the agents aligns with the linear observation model used in the recovery problem (6). This misalignment gives rise to an error that contributes to the effective residual $\epsilon_{1}$. Understanding and bounding this term is essential to guarantee a stable and accurate recovery. The following proposition quantifies the Accelerated-LVP error, thus providing a theoretical justification for choosing a valid tolerance parameter in the recovery formulation.

**Proposition 2. (Accelerated-LVP Error Estimation).** *Consider a sequence of estimates $\{{\bar{\hat{𝐲}}}_{t}{\}}_{t=0}^{\infty }$ generated by Accelerated-LVP, and assume there exists a global optimum ${\bar{𝐲}}_{t}$ of the function*

$$R_{t}(\bar{\hat{𝐲}})=\frac{\left(1+\lambda \right)}{2}{\bar{\hat{𝐲}}}^{T}({𝐈}_{m}⊗ℒ(B(t)))\bar{\hat{𝐲}}.$$ (12)

*Suppose that for each iteration t, the following inequality holds [40]:*

$${𝔼}_{t}[R_{t}({\bar{\hat{𝐲}}}_{t})-R_{t}({\bar{𝐲}}_{t})]\leq \lambda_{t}(\phi_{0}({\bar{𝐲}}_{0})-R_{t}({\bar{𝐲}}_{t})+\Phi )+D_{t},$$ (13)

*where:*

- $\phi_{0}({\bar{𝐲}}_{0})$ *is an initial surrogate function,*
- Φ is a finite constant,
- $\lambda_{t}$ *characterizes the error decay rate,*
- $D_{t}\geq 0$ *is a residual error, satisfying $D_{t}<D_{\infty }<\infty$ and $D_{t}\to D_{\infty }$ as t→∞.*

*Then, the following statements hold:*

1. **Error bound after *K* iterations.** *For any K≥1*:
  $${𝔼}_{K}[R_{K}({\bar{\hat{𝐲}}}_{K})-R_{K}({\bar{𝐲}}_{K})]\leq \lambda_{K}(\phi_{0}({\bar{𝐲}}_{0})-R_{K}({\bar{𝐲}}_{K})+\Phi )+D_{K}.$$ (14)
2. **Asymptotic error bound.** *In the limit:*
  $${lim sup}_{K\to \infty }{𝔼}_{K}[R_{K}({\bar{\hat{𝐲}}}_{K})-R_{K}({\bar{𝐲}}_{K})]\leq D_{\infty }.$$ (15)
3. **Minimum number of iterations for a given accuracy.** *If a target accuracy $\epsilon >D_{\infty }$ is required, then the minimum number of iterations $K_{\min}$ satisfies:*
  $$K_{\min}=\left\lceil \frac{\ln\left(\frac{\epsilon -D_{K}}{C}\right)}{\ln(1/\lambda )}\right\rceil ,$$ (16)
*where $C=\phi_{0}({\bar{𝐲}}_{0})-R_{K}({\bar{𝐲}}_{K})+\Phi$.*

**Proof 2. Step 1: Bounding the error after *K* iterations.**

*By assumption, we have for any iteration t:*

$${𝔼}_{t}[R_{t}({\bar{\hat{𝐲}}}_{t})-R_{t}({\bar{𝐲}}_{t})]\leq \lambda_{t}(\phi_{0}({\bar{𝐲}}_{0})-R_{t}({\bar{𝐲}}_{t})+\Phi )+D_{t}.$$ (17)

*Setting *t* = *K* directly gives the first statement of the theorem.*

**Step 2: Asymptotic error bound.**

*Since $\lambda_{t}\to 0$ and $D_{t}\to D_{\infty }$ as t→∞, we take the limit superior on both sides:*

$${lim sup}_{K\to \infty }{𝔼}_{K}[R_{K}({\bar{\hat{𝐲}}}_{K})-R_{K}({\bar{𝐲}}_{K})]\leq {lim sup}_{K\to \infty }[\lambda_{K}(\phi_{0}({\bar{𝐲}}_{0})-R_{K}({\bar{𝐲}}_{K})+\Phi )+D_{K}].$$ (18)

*Since $\lambda_{K}\to 0$ and $D_{K}\to D_{\infty }$, we conclude that*

$${lim sup}_{K\to \infty }{𝔼}_{K}[R_{K}({\bar{\hat{𝐲}}}_{K})-R_{K}({\bar{𝐲}}_{K})]\leq D_{\infty }.$$ (19)

**Step 3: Estimating the number of iterations for accuracy ϵ.**

*To satisfy the accuracy requirement:*

$${𝔼}_{K}[R_{K}({\bar{\hat{𝐲}}}_{K})-R_{K}({\bar{𝐲}}_{K})]\leq \epsilon ,$$ (20)

*we require:*

$$\lambda_{K}(\phi_{0}({\bar{𝐲}}_{0})-R_{K}({\bar{𝐲}}_{K})+\Phi )+D_{K}\leq \epsilon .$$ (21)

*Then:*

$$\lambda_{K}C\leq \epsilon -D_{K}.$$ (22)

*where $C=\phi_{0}({\bar{𝐲}}_{0})-R_{K}({\bar{𝐲}}_{K})+\Phi$. If $\lambda_{K}=\lambda^{K}$, then:*

$$\lambda^{K}C\leq \epsilon -D_{K}.$$ (23)

*Taking the logarithm on both sides:*

$$K\ln(\lambda )\leq \ln\left(\frac{\epsilon -D_{K}}{C}\right).$$ (24)

*Since ln(λ)<0, dividing by it reverses the inequality:*

$$K\geq \frac{\ln\left(\frac{\epsilon -D_{K}}{C}\right)}{\ln(1/\lambda )}.$$ (25)

*Thus, the minimum number of iterations is:*

$$K_{\min}=\left\lceil \frac{\ln\left(\frac{\epsilon -D_{K}}{C}\right)}{\ln(1/\lambda )}\right\rceil .$$ (26)

*This completes the proof.*

Traditional methods for determining cluster centroids, such as interior-point methods, are computationally expensive and unsuitable for agents with limited processing power. We propose a novel approach to overcome this limitation: pre-training a neural network on a diverse set of possible or likely scenarios. Instead of directly performing complex computations, agents can access a service that efficiently delivers the output of this pre-trained neural network. The following subsection details the reconstruction process using this neural network.

### Reconstruction via a neural network

In compressed sensing, signal reconstruction typically involves solving an optimization problem to recover a sparse signal $\tilde{𝐱}$ (where most components are zero or near– zero) from a set of measurements 𝐲. Traditionally, neural networks can learn to map these under-sampled measurements 𝐲 back to the original signal $\tilde{𝐱}$. Our approach differs from this traditional method. First, each agent aggregates its local measurements ${𝐲}_{i}$ using the Accelerated-LVP. Second, instead of reconstructing the original signals $\tilde{𝐱}$, we focus solely on reconstructing the cluster centroids.

A typical deep learning-based reconstruction process can be formulated as learning a mapping function $f_{\theta }$ such that:


$$\hat{\Theta }=f_{\theta }(𝐲)$$

where $\hat{\Theta }$ represents the estimated positions of the cluster centroids ${\tilde{\mu }}_{l}$, for $l=1,\ldots ,n_{c}$, along with the corresponding cluster magnitudes $\left|{𝒞}_{l}\right|$.

The actual network architecture is as follows:

1. Input Layer: Linear(compressed size, 512)
  - Fully connected layer mapping input to 512 dimensions.
2. Hidden Layers:
  - Layer 1:
    - Linear(512, 512)
    - ReLU()
    - BatchNorm1d(512)
  - Layer 2:
    - Linear(512, 512)
    - ReLU()
    - BatchNorm1d(512)
  - Layer 3:
    - Linear(512, 256)
    - ReLU()
    - BatchNorm1d(256)
  - Layer 4:
    - Linear(256, 128)
    - ReLU()
3. Output Layer:
  - Linear(128, 2 × clusters_number)
  - Reshape with Unflatten(1, (2, clusters_number)) to create a (2, clusters_number) output structure.

The neural network receives the under-sampled measurement $\bar{𝐲}$ as input and produces an estimate of the centroids $\hat{\Theta }$ as output. The Hungarian Loss is applied to calculate the overall *L*_2_ difference (like [41]) between the predicted centroids and the ground-truth centroids based on the optimal clusters’ assignment provided by the Hungarian algorithm. Standard Adam optimization is employed to train the network.

At its core, the application of neural networks in compressed sensing revolves around replacing conventional optimization or greedy algorithms with deep learning models. These models are specifically trained to discern the complex relationship between under-sampled measurements and the original sparse data. These networks effectively learn this intricate mapping by employing end-to-end training on datasets consisting of measurements paired with their corresponding ground truth signals. As a result, once trained, they offer a rapid and efficient approach for reconstructing signals within the framework of compressed sensing applications.

Compressed sensing theory provides guarantees for recovering sparse signals via $\ell_{1}$-optimization, provided that the measurement matrix satisfies the RIP. In the absence of noise, this condition ensures that $\ell_{1}$-minimization yields a unique sparsest solution. The reconstruction process can be viewed as a mapping, allowing us to define a function *f* that maps compressed measurements to the recovered sparse signal. The RIP condition implies that *f* is continuous, meaning that small changes in the measurement vector $\bar{𝐲}$ result in correspondingly small deviations in the reconstructed signal $\tilde{𝐱}$. By the Universal Approximation Theorem, such a continuous function *f* can be approximated by a neural network $f_{\theta }$ parameterized by θ, offering an efficient and learnable approach to sparse signal recovery from compressed data.

### Proposed algorithm

To conclude, we outline our proposed method in Algorithm 2 being a combination of three consecutive steps:

**Step 1: Sampling to obtain a Sparse State**

Each agent quantizes its continuous state space by mapping it onto a discrete grid ensuring the data is sparse enough for effective representation, while capturing critical features, such as cluster centers. The procedure preserves the signal’s integrity using a sufficiently fine grid, facilitating accurate downstream processing. This discretization can be considered as a preliminary transformation to prepare the state for efficient compression.

**Step 2: CS-Based Compression via Accelerated-LVP Aggregation**

In this step, the compressed sensing principles are applied. Each agent compresses its grid-discretized state using a Gaussian measurement matrix. The CS technique capitalizes on the sparsity of the grid representation, significantly reducing data dimensionality while retaining the most relevant information. The resulted compressed portrayal serves as a reduced-dimensional stand-in for the global state, which can be used for further processing or reconstruction tasks.

In the context of aggregation, where the goal is to aggregate local measurements from multiple agents, we use accelerated optimization methods, i.e. Accelerated-LVP, to efficiently combine these measurements. The idea is to take advantage of the faster convergence of accelerated consensus methods to aggregate data quickly and accurately.

**Step 3: Reconstruction via a neural network**

Finally, we employ neural networks to reconstruct the centroids from the directly aggregated compressed measurements.

**Algorithm 2 Decentralized clustering using compressed sensing and neural network.**

**Inputs:**

- Agent local states ${𝐱}_{i}\in {\mathbb{R}}^{d}$

- Measurement matrix $\Phi \in {\mathbb{R}}^{m\times g_{s}}$

- Communication topology 𝒢

- Number of local voting protocol iterations $n_{lvp}$

- Parameters of Accelerated-LVP: $𝒫=\{h,L,\mu ,\eta ,\alpha_{x},\gamma ,\lambda \}$

**Output:** Cluster assignment for each agent *i*

**Training Stage:**

1: Generate synthetic dataset (states and centroids)

2: Map states ${𝐱}_{i}$ *to* grid cells to obtain common vector $\tilde{\text{X}}$

3: Compute compressed measurements $\tilde{\text{Y}}=\Phi \tilde{\text{X}}$

4: Train neural network $f_{\theta }$ *to* find a set of centroids Θ *based*

on $\frac{1}{\sqrt{n}}\tilde{\text{Y}}$

**Inference Stage:**

1: **For** each agent *i* **do**

2:   Obtain local state ${𝐱}_{i}$

3:   Map ${𝐱}_{i}$ *to* grid cell and obtain representation ${\tilde{\text{X}}}_{i}$

4:   Get local measurement ${𝐲}_{i}$

5:   Initialize aggregated measurement ${\hat{𝐲}}_{i}\left(0\right)={𝐲}_{i}$

6: **end for**

7: **for** each agent *i* **do**

8:   Update aggregated measurement using Accelerated-LVP

(Algorithm 1) with parameters $𝒫=\{h,L,\mu ,\eta ,\alpha_{x},\gamma ,𝒢,\lambda \}$

9: **end for**

10: **for** each agent *i* **do**

11:   Find centroids set from aggregated compressed measurement

${\hat{𝐲}}_{i}\left(n_{lvp}\right)$:


$${\hat{\Theta }}_{i}=f_{\theta }({\hat{𝐲}}_{i}\left(n_{lvp}\right))$$

12:   Determine cluster assignment:


$$l^{*}=\arg\min_{l}\|{𝐱}_{i}-{\hat{\theta }}_{l}{\|}_{2}$$

13: **end for**

The proposed algorithm employs several strategies to accelerate the overall process:

- **Leveraging Reduced-Dimensional Representations of States:** Using lower-dimensional representations of the states substantially reduces the computational complexity of processing high-dimensional data. This facilitates more efficient data exchange and aggregation between agents, accelerating the convergence process.
- **Applying Accelerated Optimization During Aggregation:** During the aggregation step, accelerated optimization techniques are incorporated to speed up the convergence of the aggregation process improving the optimization efficiency, reducing the number of iterations required for consensus and enabling faster communication between agents.
- **Directly Restoring Centroids Using Pre-Trained Neural Networks:** The approach centers on reconstructing only the compact centroids that effectively represent the global estimate. By leveraging pre-trained neural networks for this purpose, traditional optimization methods are bypassed, enabling a highly efficient reconstruction process. These models efficiently map the aggregated measurements to the centroids, delivering a significant speed-up compared to conventional reconstruction techniques.

Generally, this combined approach not only reduces dimensionality and computational costs at each step but also accelerates aggregation and reconstruction, leading to a more efficient and scalable algorithm. Moreover, unlike traditional decentralized approaches that may require direct data sharing, our method ensures that individual sensor data is never transmitted between agents. Instead, nodes exchange only aggregated clustering-related information, significantly reducing privacy risks.

#### Analysis of the proposed algorithm.

Our analysis focuses on studying the impact of input errors in a neural network. These errors arise from inaccuracies in recovering the cluster structure and from the data aggregation procedure (see (8)).

Since input noise is considered in a neural network, the total network error will depend on several factors:

1. **Model Error** ($\varepsilon_{\text{model}}$): The error related to the architecture of the network, its parameters, and its ability to approximate the true function.
2. **Training Error** ($\varepsilon_{\text{train}}$): The error caused by a limited training dataset, insufficient optimization of weights, overfitting, or underfitting.
3. **Input Error** ($\varepsilon_{\text{input}}$): The error associated with noise or inaccuracy in the input data, i.e., $\epsilon_{1}$ and $\epsilon_{2}$ in Eq (8).

Thus, the **total error** of the network is:

$$\varepsilon_{\text{total}}=\varepsilon_{\text{model}}+\varepsilon_{\text{train}}+\varepsilon_{\text{input}}.$$ (27)

We then explore the behavior of neural networks under input noise. First, we introduce an assumption regarding the neural network.

**Assumption 4. (Lipschitz neural network).** *The considered neural network $f_{\theta }:{\mathbb{R}}^{m}\to {\mathbb{R}}^{n_{c}}$ is supposed to be γ-Lipschitz continuous with respect to its input, i.e.,*


$$\|f_{\theta }(x)-f_{\theta }(y)\|\leq \gamma \|x-y\|,\;\text{for all }x,y\in {\mathbb{R}}^{m}.$$

Note that for a target function $f^{*}$, the total error can be decomposed as:


$$\|f_{\theta }(x+\delta x)-f^{*}(x){\|}^{2}\leq \underset{\text{Model + Training Error}}{\underbrace{\|f_{\theta }(x)-f^{*}(x){\|}^{2}}}+\underset{\text{Input Noise Error}}{\underbrace{\left\|f_{\theta }(x+\delta x)-f_{\theta }(x)\right\|}}.$$

**Proposition 3. (Input Noise Propagation under Lipschitz Networks).** *Consider a base input $x\in {\mathbb{R}}^{m}\text{ and an additive noise or perturbation}\delta x$, so that the actual (perturbed) input is x+δx. Under Assumption 4, we have:*

1. **Deterministic noise.** *If δx is a bounded, deterministic perturbation with $\|\delta x\|\leq \Delta_{x}$, then*
  $$\|f_{\theta }(x+\delta x)-f_{\theta }(x)\|\leq \gamma \|\delta x\|\leq \gamma \Delta_{x}.$$
2. **Random noise.** *If $\delta x\text{ is a zero}-\text{mean random variable with }𝔼[\delta x]=0\text{ and covariance }\Sigma_{x}=𝔼[\delta x\delta x^{⊤}]$, then*
  $$𝔼\left[\|f_{\theta }(x+\delta x)-f_{\theta }(x){\|}^{2}\right]\leq \gamma^{2}\;𝔼[\|\delta x{\|}^{2}]=\gamma^{2}\;Tr(\Sigma_{x}).$$

**Proof 3.** *1. Deterministic Noise Case*

*For a target function *f** ^*^ *, we have*


$$\|f_{\theta }(x+\delta x)-f^{*}(x)\|\leq \|f_{\theta }(x+\delta x)-f_{\theta }(x)\|+\|f_{\theta }(x)-f^{*}(x)\|\;\leq \gamma \|\delta x\|+\|f_{\theta }(x)-f^{*}(x)\|.$$

*By Lipschitz continuity:*


$$\|f_{\theta }(x+\delta x)-f_{\theta }(x)\|\leq \gamma \|\delta x\|.$$

*Since $\|\delta x\|\leq \Delta_{x}$, we obtain*


$$\|f_{\theta }(x+\delta x)-f_{\theta }(x)\|\leq \gamma \Delta_{x}.$$

*2. Random Noise Case*

*From Lipschitz continuity:*


$$\|f_{\theta }(x+\delta x)-f_{\theta }(x)\|\leq \gamma \|\delta x\|\;\Rightarrow \;\|f_{\theta }(x+\delta x)-f_{\theta }(x){\|}^{2}\leq \gamma^{2}\|\delta x{\|}^{2}.$$

*Taking the expectation on both sides, we derive:*


$$𝔼[\|f_{\theta }(x+\delta x)-f_{\theta }(x){\|}^{2}]\leq \gamma^{2}𝔼[\|\delta x{\|}^{2}].$$

*Since $𝔼[\|\delta x{\|}^{2}]=Tr(\Sigma_{x})$, we get:*


$$𝔼[\|f_{\theta }(x+\delta x)-f_{\theta }(x){\|}^{2}]\leq \gamma^{2}\;Tr(\Sigma_{x}).$$

*Then the total error satisfies:*


$$𝔼[\|f_{\theta }(x+\delta x)-f^{*}(x){\|}^{2}]\leq 𝔼[\|f_{\theta }(x)-f^{*}(x){\|}^{2}]+\gamma^{2}Tr(\Sigma_{x}).$$

*This completes the proof.*

## Simulations

### Datasets

To evaluate the performance and robustness of the proposed approach, we conduct experiments on two types of datasets: one synthetic (artificial) and one derived from real-world observations. The synthetic dataset allows for controlled evaluation under known ground-truth coalition structures, while the real-world dataset demonstrates the method’s applicability in practical environments.

**Synthetic dataset.** The centroids $\theta_{l}$ for each cluster *l* are generated from a uniform distribution:


$$\theta_{l}\sim U([0,1]\times [0,1]),\;l=1,2,\ldots ,n_{c}$$

The number of points *m*_*l*_ for each cluster *l* is generated from a multinomial distribution with equal probabilities, ensuring a total of 3000 points for all clusters:


$$\left(m_{1},m_{2},\ldots ,m_{n_{c}})\sim \text{\{Multinomial}\}(3000,\frac{1}{n_{c}},\ldots ,\frac{1}{n_{c}}\right)$$

Points within each cluster are drawn from a multivariate normal distribution:


$$\text{\{Cluster }\}l:\;p\sim 𝒩(\theta_{l}+\epsilon_{l},\Sigma_{l}),$$

where $\epsilon_{l}$ is a small perturbation, and the covariance matrix $\Sigma_{l}$ is generated from a uniform distribution (with standard deviations in [0.001,0.01]).

**Real-world evaluation.** We use the GeoLife GPS Trajectory Dataset developed by Microsoft Research Asia [42]. This dataset contains over 17,000 GPS trajectories collected from 182 users over a period of five years. The data was recorded with varying sampling rates and contains rich spatiotemporal information including latitude, longitude, altitude, and timestamp. GeoLife captures real human mobility patterns such as commuting, biking, and walking, making it a relevant and challenging benchmark for evaluating decentralized algorithms in location-aware and mobility-driven systems.

### Baseline and metrics

We compare the method with the baseline distributed k-means algorithm presented in [7]. This method represents a strong baseline, particularly for distributed clustering under communication constraints.

We use the following metrics to compare the proposed algorithm with the baseline:

- **Adjusted Rand Index (ARI)** measures the similarity between the predicted clusters assignments and the actual labels, adjusted for the chance. A value of 1 indicates perfect agreement.
- **Normalized Mutual Information (NMI)** quantifies the mutual information between the predicted clusters and the actual labels, normalized to ensure values between 0 and 1, where 1 represents perfect agreement.
- **Silhouette Score** assesses how well-separated clusters are, with values ranging from -1 to 1, where higher values indicate better clustering.
- **Mean Absolute Error (MAE)** measures the average absolute error between the predicted and true cluster centroids.
- **Dunn Index** evaluates clustering quality by taking the ratio between the smallest distance between cluster centroids and the largest intra-cluster distance. Higher values reflect compact and well-separated clusters.
- **Davies–Bouldin Index (DBI)** assesses clustering validity based on the average similarity between each cluster and its most similar counterpart. Lower values indicate better separation and compactness.
- **Communication Cost** is the total number of exchanged messages across all nodes.

**Remark 3.** *Notably, many existing methods, see, e.g., [5], are designed for scenarios with a relatively small number of agents, each having large local datasets. In contrast, our focus is on a setting where there is a large number of agents, but each possesses very limited local data. This fundamental difference makes direct comparisons with some state-of-the-art methods less applicable, as they rely on richer local data for effective clustering.*

### Experimental validation using synthetic data

The experiments are carried out with a batch size of 128. The Adam optimizer is used with an initial learning rate of 0.003, which linearly decreases to 0.0003. The model is trained on 1,000,000 samples and tested on a validation set of 10,000 samples. The setup involves point clouds from 1,000 agents mapped onto a 128×128 grid, clustered into 10 clusters and 3 clusters. The source code is available here: https://github.com/vkpankov/cs_decentralized_clustering.

**Fig 1:**
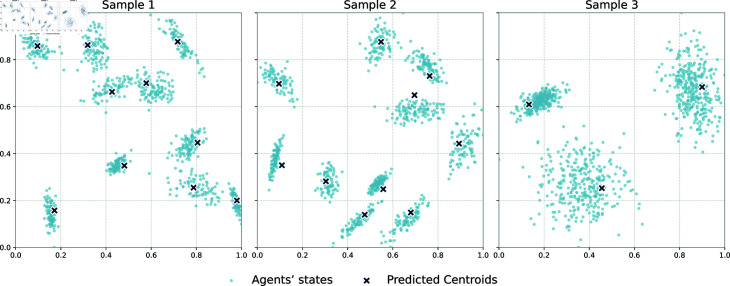
Examples of three random samples from the validation dataset. Each sample shows 1000 agents’ states mapped onto a 128×128 grid (normalized to [0,1]×[0,1]), together with the predicted centroids: 10 centroids for Samples 1 and 2, and 3 centroids for Sample 3.

Random samples are shown in Fig 1.

**Impact of consensus procedure on the reconstruction quality.** We perform distributed reconstruction of 10 clusters with simulated distributed compressed sensing, employing the Accelerated-LVP with step size $h=0.01\;and\;binary\;weights\;b_{i,j}$. A random connectivity matrix *B*_*t*_ is utilized, in which two agents have 2% probability of being connected. As illustrated in Tables 1 and 2, the precision of the reconstruction increases with the number of iterations.

**Table 1:** Summary of clustering metrics across Accelerated-LVP iterations. Part I.

Num it	ARI	NMI	Silh. Score
5	0.803±0.081	0.896±0.036	0.573±0.083
7	0.855±0.100	0.917±0.050	0.613±0.100
10	0.887±0.061	0.933±0.032	0.635±0.064
15	0.883±0.064	0.930±0.034	0.626±0.074
20	0.902±0.073	0.945±0.036	0.668±0.072
30	0.919±0.080	0.949±0.047	0.659±0.080

**Table 2:** Summary of clustering metrics across Accelerated-LVP iterations. Part II.

Num it	MAE	Comm. Cost
5	11.871±3.373	3223832
7	7.933±3.523	4858687
10	7.084±1.903	7296524
15	6.595±1.788	11303371
20	5.988±2.146	15399234
30	5.885±2.001	23455469

**Fig 2:**
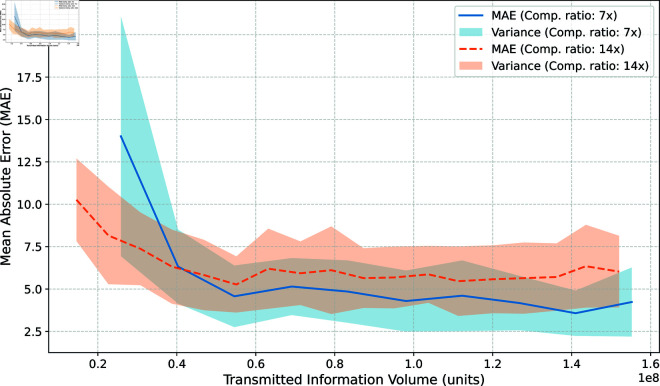
Effect of compression on reconstruction quality under random communication topology. The plot shows the relationship between clustering quality (measured by relative mean absolute error between predicted and ground-truth centroids) and the total transmitted information volume across all agents. Two compression modes are evaluated: 14x and 7x. The shaded areas represent variance across 30 runs with random topologies, where each pair of agents has a 0.02 probability of being connected.

**Impact of compression on the reconstruction quality.** In this experiment, we evaluate the reconstruction quality using the mean absolute error. We consider two compression modes: 14x and 7x compression, where the compressed signal is 14 times and 7 times smaller than the original signal, respectively. The communication topology is random, with a 0.02 probability that any two agents are connected. Fig 2 illustrates the relationship between clustering quality (measured by the relative MAE between predicted and ground-truth centroids) and the total volume of transmitted information across all agents. The shaded region around the curves indicates the variance observed across 30 runs with random topologies.

**Comparison with the baseline.** Next, we compare our method with the k-means-distributed baseline algorithm, aligning their communication costs to isolate and compare reconstruction accuracy. The communication cost differs by approximately 2%. Table 3 presents a comprehensive comparison of the proposed method with the distributed baseline [7] and the centralized ground-truth clustering (k-means). The proposed method demonstrates consistently superior clustering performance compared to the distributed baseline across all evaluation metrics, and it closely approaches the performance of the centralized ground-truth solution. Importantly, all differences between the proposed and distributed methods are statistically significant (p<0.01) based on paired *t*-tests.

**Table 3:** Comparison of clustering performance metrics for the proposed method, distributed baseline [7], and ground-truth (centralized k-means). Mean and standard deviation values are reported. Statistically significant differences (p < 0.01) were observed between the proposed and distributed methods using a paired t-test.

Metric	Proposed	Distributed KM	Centralized KM	p-val
ARI (↑)	0.900 ± 0.075	0.734 ± 0.105	0.994 ± 0.007	0.000
NMI (↑)	0.940 ± 0.042	0.877 ± 0.048	0.994 ± 0.007	0.000
Silhouette (↑)	0.648 ± 0.072	0.556 ± 0.095	0.703 ± 0.042	0.003
Dunn Index (↑)	0.021 ± 0.012	0.008 ± 0.005	0.053 ± 0.032	0.001
Davies-Bouldin (↓)	0.511 ± 0.135	0.672 ± 0.172	0.403 ± 0.050	0.004
MAE (↓)	5.972 ± 1.615	11.107 ± 2.455	0.837 ± 0.224	0.000
Comm. Cost (↓)	8.3M	9.5M	-	-

The proposed method achieves high clustering accuracy, with the Adjusted rand index (ARI) is about 0.900 and the Normalized mutual information (NMI) of 0.940. These scores are significantly higher than those of the distributed baseline (ARI: 0.734, NMI: 0.877), and closely approach those of the centralized k-means solution (ARI and NMI: 0.994), indicating strong agreement with the true labels.

The proposed method also performs better in terms of structural metrics. It achieves a higher Silhouette Score (0.648 vs. 0.556) and Dunn Index (0.021 vs. 0.008), reflecting improved intra-cluster compactness and inter-cluster separation. Additionally, it yields a lower Davies–Bouldin Index (0.511 vs. 0.672), suggesting more coherent and distinct clusters. Although centralized k-means still performs best on these metrics, the gap is significantly reduced.

The Mean Absolute Error (MAE) between predicted and true cluster centroids is substantially lower for the proposed method (5.972) compared to the distributed baseline (11.107), demonstrating enhanced precision in centroid estimation. Centralized k-means achieves near-optimal MAE (0.837), but assumes full data centralization.

The proposed method also demonstrates improved communication efficiency, requiring only 8.3 million messages compared to 9.5 million for the distributed baseline. This reduction highlights the method’s scalability and practicality in decentralized settings where bandwidth is limited.

**Ablation study.** We also conducted experiments with alternative backbone architectures, all of which demonstrated notably lower performance compared to our proposed method. For example, a Transformer encoder with 6 layers, 4 attention heads, and a head dimension of 512 was trained on linearly projected compressed measurements for four times the number of iterations used in our model. Despite the extended training, the Transformer consistently underperformed across all clustering metrics (see Table 4). Additionally, we explored a diffusion-based method for reconstructing cluster positions and object detection-inspired approaches that treated the input as an image to identify the centers and sizes of ellipse-shaped clusters. These methods also failed to match the effectiveness of our approach.

**Table 4:** Ablation study results.

Metric	Proposed	Transformer-Based
ARI (↑)	0.900 ± 0.075	0.662 ± 0.068
NMI (↑)	0.940 ± 0.042	0.824 ± 0.044
Silhouette (↑)	0.648 ± 0.072	0.447 ± 0.110
Dunn Index (↑)	0.021 ± 0.012	0.007 ± 0.003
Davies-Bouldin (↓)	0.511 ± 0.135	0.753 ± 0.192
MAE (↓)	5.972 ± 1.615	19.950 ± 1.612

**Fig 3:**
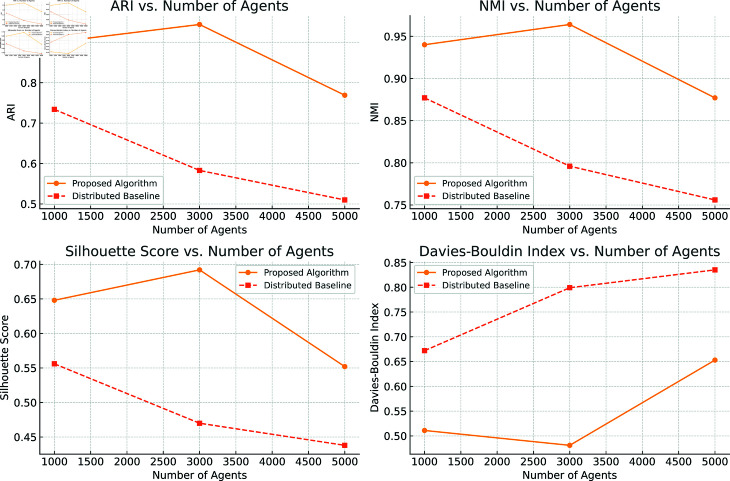
Evaluation of the proposed algorithm across varying agent counts (1,000, 3,000, and 5,000), using four metrics: Adjusted Rand Index (ARI), Normalized Mutual Information (NMI), Silhouette Score, and Davies-Bouldin Index. Each metric is plotted against the number of agents. The proposed method demonstrates consistently superior performance compared to the baseline distributed approach. A slight degradation in clustering quality at 5,000 agents indicates potential scalability limitations, which may be addressed through architectural modifications such as increased network depth or additional internal channels.

**Scalability under varying agent counts.** Fig 3 illustrates the metrics of our decentralized clustering algorithm evaluated with 1000, 3000, and 5000 agents. The overall clustering quality remains high across all tested configurations and consistently outperforms the baseline distributed method. A moderate decrease is observed at 5000 agents, suggesting that in large-scale scenarios, some architectural changes—such as increasing the network depth or the number of internal channels—should be explored to maintain quality.

### Experimental validation using real-world data

Table 5 presents the performance metrics obtained from real-world experiments conducted on the GeoLife GPS trajectory dataset [42]. In each of the 20 runs, 3,000 data points were randomly sampled from the densest subregion of the dataset, defined by normalized longitude in the range [0.3, 0.5] and latitude in [0.5, 0.6]. The results compare the proposed method against both the distributed baseline and the centralized k-means algorithm.

**Table 5:** Real-world metrics.

Metric	Proposed	Distributed	Centralized	p-val
Silhouette (↑)	0.410 ± 0.003	0.279 ± 0.042	0.482 ± 0.004	0.000
Dunn Index (↑)	0.003 ± 0.001	0.002 ± 0.001	0.004 ± 0.001	0.003
Davies-Bouldin (↓)	0.875 ± 0.004	1.111 ± 0.074	0.741 ± 0.005	0.000

The results from the real-world evaluation confirm the effectiveness of the proposed method in producing well-structured clusters under decentralized conditions. The proposed algorithm consistently outperforms the distributed baseline across all structure-based clustering metrics, with all improvements being statistically significant (p<0.01).

The Silhouette score improves from 0.279 (distributed baseline) to 0.410, indicating better-defined and more coherent clusters. Although the centralized k-means still achieves the highest score (0.482), the proposed method substantially narrows the gap. The Dunn index also increases slightly from 0.002 to 0.003, suggesting improved inter-cluster separation and intra-cluster compactness relative to the baseline. The Davies–Bouldin index is reduced from 1.111 to 0.875, reflecting a more compact well-separated cluster configuration. While the centralized approach yields the lowest value (0.741), the proposed method offers a significant improvement over the baseline in a decentralized setting.

Overall, these findings demonstrate that the proposed method maintains strong performance in real-world environments and achieves a good balance between clustering quality and decentralized applicability.

## Discussion

This work presents a decentralized clustering method that utilizes compressed states for node communication. The results emphasize the effectiveness of this approach in reducing communication overhead and ensuring efficient operation in environments with limited computational resources.

**Communication Efficiency:** One of the key advantages of our method is the significant reduction in the amount of data exchanged between nodes. By using compressed states for communication, we limit the transmission of raw data, which can be costly in terms of bandwidth, especially in environments with many nodes or constrained resources. Our results show that the compression technique does not compromise the quality of the clustering process, making it a highly efficient solution for decentralized systems. In fact, the compressed measurements allow the agents to operate collaboratively while transmitting only essential information.

**Computational Efficiency:** Another significant benefit of our method lies in its computational efficiency. By using a pre-trained neural network to handle the complex computations required for clustering, agents with limited processing power can still contribute effectively to the overall task. The use of a neural network that has been trained on a variety of scenarios reduces the need for agents to perform resource-intensive calculations locally. This aspect of the approach ensures that agents with low computational resources can participate in the clustering process without being bottlenecks. The computational savings offered by the neural network are a crucial advantage, allowing the system to scale more effectively without overwhelming individual agents. The proposed algorithm achieves fast inference, requiring only 0.465±0.006 ms to predict cluster assignments from compressed measurements on an Nvidia RTX 3080 paired with an AMD Ryzen 3990X. In contrast, centralized k-means clustering on the full dataset is considerably slower, with an average runtime of 133.4±10.092 ms.

**Performance Comparison:** When comparing our decentralized clustering approach to the baseline method, we found that our method outperforms it in terms of accuracy. Specifically, we observe a 31.5% increase in the ARI metric, an 11.73% improvement in the NMI metric, a 30% rise in the Silhouette Score, and a 48.4% reduction in the Mean Absolute Error. This suggests that the use of compressed states for communication does not significantly compromise the quality of the results, and the system can achieve reliable clustering performance while minimizing the data exchange between nodes.

**Practical Implementation and Model Limitations:** While the proposed method performs well within the tested conditions, limitations warrant further investigation. A challenge in the current framework is the need to dynamically evaluate the actual number of clusters. This can be addressed through a two-stage approach: first, estimating the most likely number of clusters, followed by validating and correcting the resulting partition.

Another challenge in high-dimensional agent states is the inefficiency of grid-based discretization, which motivates the use of learned point representations or sparse quantization methods that scale more effectively with dimensionality. Crucially, these methods must remain compatible with the distributed communication model and compression pipeline, particularly the ability to express representations as aggregations across agents. The compression method’s effectiveness hinges on input data features. In highly variable or noisy environments, the method might degrade important information, potentially harming clustering quality. Adaptive compression schemes, designed for specific data characteristics or network conditions, could enhance robustness.

It is important to note that the pre-trained neural network’s performance is tied to the diversity of its training data. Although some robustness to out-of-distribution scenarios is observed, practical deployments should integrate synthetic data with domain-specific priors.

## Conclusion and future research

In conclusion, this work introduces a decentralized clustering method using compressed states for communication, improving both communication and computational efficiency. By reducing the amount of data exchanged between nodes, the approach minimizes communication overhead, which is especially valuable in resource-constrained environments.

The method also applies a pre-trained neural network to handle complex computations, allowing agents with limited resources to contribute effectively without becoming bottlenecks. In terms of performance, the proposed method outperforms the baseline with a 31.5% increase in ARI, an 11.73% improvement in NMI, a 30% rise in Silhouette Score, and a 48.4% reduction in Mean Absolute Error, demonstrating high-quality clustering with reduced resource demands.

Although the presented method focuses on centroid recovery, a potential area for future development is the reconstruction of richer structural features, such as cluster shapes or densities. Compressed representations may encode more information than is currently utilized, opening possibilities for more detailed structural analysis. Future work could be devoted to such a development, combined with optimizing the compression technique and exploring adaptive neural network models.
